# Coexistence of the Entner–Doudoroff and Embden–Meyerhof–Parnas pathways enhances glucose consumption of ethanol-producing *Corynebacterium glutamicum*

**DOI:** 10.1186/s13068-021-01876-3

**Published:** 2021-02-16

**Authors:** Toru Jojima, Takafumi Igari, Ryoji Noburyu, Akira Watanabe, Masako Suda, Masayuki Inui

**Affiliations:** 1 Research Institute of Innovative Technology for the Earth, 9-2, Kizugawadai, Kizugawa, Kyoto, 619-0292 Japan; 2 Division of Biological Sciences, Nara Institute of Science and Technology, Takayama, Ikoma, 8916-5, Nara, 630-0101 Japan; 3 Present Address: Faculty of Agriculture, Department of Environmental Management, Kindai University, 3327-204 Nakamachi, Nara, 631-8505 Japan

**Keywords:** *Corynebacterium glutamicum*, Glycolysis, Entner–Doudoroff pathway, Oxygen deprivation

## Abstract

**Background:**

It is interesting to modify sugar metabolic pathways to improve the productivity of biocatalysts that convert sugars to value-added products. However, this attempt often fails due to the tight control of the sugar metabolic pathways. Recently, activation of the Entner–Doudoroff (ED) pathway in *Escherichia coli* has been shown to enhance glucose consumption, though the mechanism underlying this phenomenon is poorly understood. In the present study, we investigated the effect of a functional ED pathway in metabolically engineered *Corynebacterium glutamicum* that metabolizes glucose via the Embden–Meyerhof–Parnas (EMP) pathway to produce ethanol under oxygen deprivation. This study aims to provide further information on metabolic engineering strategies that allow the Entner–Doudoroff and Embden–Meyerhof–Parnas pathways to coexist.

**Results:**

Three genes (*zwf*, *edd*, and *eda*) encoding glucose-6-phosphate dehydrogenase, 6-phosphogluconate dehydratase, and 2-keto-3-deoxy-6-phosphogluconate aldolase from *Zymomonas mobilis* were expressed in a genetically modified strain, *C. glutamicum* CRZ2e, which produces pyruvate decarboxylase and alcohol dehydrogenase from *Z. mobilis*. A ^13^C-labeling experiment using [1-^13^C] glucose indicated a distinctive ^13^C distribution of ethanol between the parental and the ED-introduced strains, which suggested an alteration of carbon flux as a consequence of ED pathway introduction. The ED-introduced strain, CRZ2e-ED, consumed glucose 1.5-fold faster than the parental strain. A *pfkA* deletion mutant of CRZ2e-ED (CRZ2e-EDΔ*pfkA*) was also constructed to evaluate the effects of EMP pathway inactivation, which showed an almost identical rate of glucose consumption compared to that of the parental CRZ2e strain. The introduction of the ED pathway did not alter the intracellular NADH/NAD^+^ ratio, whereas it resulted in a slight increase in the ATP/ADP ratio. The recombinant strains with simultaneous overexpression of the genes for the EMP and ED pathways exhibited the highest ethanol productivity among all *C. glutamicum* strains ever constructed.

**Conclusions:**

The increased sugar consumption observed in ED-introduced strains was not a consequence of cofactor balance alterations, but rather the crucial coexistence of two active glycolytic pathways for enhanced glucose consumption. Coexistence of the ED and EMP pathways is a good strategy for improving biocatalyst productivity even when NADPH supply is not a limiting factor for fermentation.

## Background

Modifying cell metabolism to produce valuable molecules is of substantial economic and scientific interest. Most of these efforts are directed toward the expression of heterologous biosynthetic pathways, inactivation of enzymes to repress by-product formation, and enhancement of the expression of enzymes specifically related to product biosynthesis [1]. All these approaches have produced valuable information on the metabolic engineering of microorganisms. In contrast, although the engineering of sugar metabolism pathways, including the Embden–Meyerhof–Parnas (EMP) pathway to increase the productivity of target products, has been of interest owing to the crucial role in sugar-based microbial cell factories, it has been less successful to date [2]. In particular, *Saccharomyces cerevisiae* has been used to study the overexpression of genes encoding glycolytic enzymes; however, almost no positive effect on ethanol productivity has been observed [3, 4].

Contrary to the above reports on *S. cerevisiae*, we found that the rates of sugar consumption and product formation were largely enhanced by the overexpression of some glycolytic genes including *pfkA, tpi* and *gapA* in *Corynebacterium glutamicum* under conditions of oxygen deprivation [2, 5, 6]. *C. glutamicum* is a Gram-positive bacterium and it has been industrially used in the production of amino acids, including l-glutamate and l-lysine, for several decades [7]. The processes for amino acid production by *C. glutamicum* are performed under aerobic conditions; however, this microorganism can also metabolize glucose and produce mixed organic acids under oxygen-deprived conditions, although its growth is suppressed under the circumstances [8]. These properties of *C. glutamicum* have been exploited to develop unique bioprocesses that decouple cell growth from biofuel and biochemical production [9].

Previous studies on the regulation of sugar metabolism in *C. glutamicum* under conditions of oxygen deprivation showed that the intracellular redox state is a crucial factor that controls sugar consumption by *C. glutamicum* [8, 10–12]. Inefficient regeneration of NAD^+^ results in a high intracellular NADH/NAD^+^ ratio, which inhibits GAPDH and consequently slows down the sugar consumption of *C. glutamicum* under conditions of oxygen deprivation. Since inefficient sugar consumption of biocatalysts results in low productivity in sugar-based fermentation processes, fast sugar consumption is always a desirable characteristic of every biocatalyst. To this end, the production of a range of chemicals, such as amino acids [6, 11], d-lactate [13], and ethanol [14], has been successfully improved by exploring the overexpression of glycolytic genes in *C. glutamicum*.

**Fig. 1:**
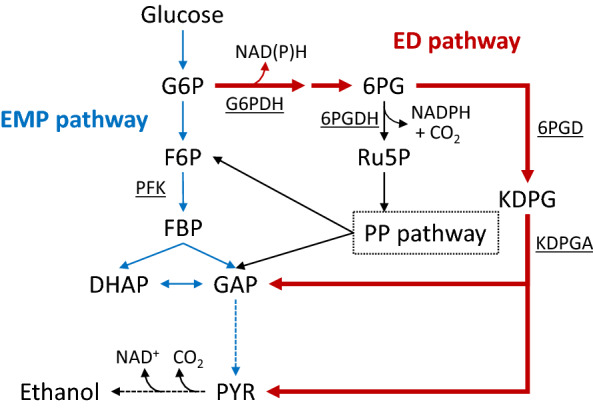
Metabolic map for the integration of the ED pathway in ethanol-producing *C. glutamicum*. Enzyme names are underlined. The ED pathway is represented by bold arrows

Recently, activation of the Entner–Doudoroff (ED) pathway in genetically engineered strains of *Escherichia coli* has been shown to enhance glucose consumption, and isopropanol [15], mevalonate [16], and isobutanol [17] production. In these studies, the ED pathway that can supply NADPH was considered to be a better pathway in terms of carbon yield than the pentose phosphate (PP) pathway that can also supply NADPH, since the generation of NADPH in the PP pathway is accompanied by CO_2_ generation in the 6-phosphogluconate dehydrogenase (6PGDH) reaction (Fig. 1). It was also expected to bypass the rate-limiting steps of the EMP pathway, including the reactions catalyzed by 6-phosphofructokinase (PFK) and fructose-bisphosphate aldolase [16]. Although these studies have clearly indicated that activation of the ED pathway enhances glucose consumption, the mechanism underlying this phenomenon is poorly understood. At least two effects induced by activation of the ED pathway, including reinforcement of NADPH supply and construction of a bypass route for sugar metabolism, might be involved in increased sugar consumption; however, the extent of their effect on the rate of glucose metabolism is unclear. Furthermore, the ED pathway has also attracted attention in the research field of fermentation, since *Zymomonas mobilis*, which metabolizes sugars via the ED pathway, consumes glucose rapidly to produce ethanol [18]. In the ED pathway, the ATP yield from one sugar molecule differs from that of the EMP pathway. Since ATP is an essential cofactor involved in the control of the EMP pathway, introduction of the ED pathway possibly affects the rate of glycolysis through the EMP pathway.

In the present study, we investigated the effect of a functional ED pathway in *C. glutamicum*, which decomposes sugar via the EMP pathway under oxygen deprivation [8, 19, 20], and the influence of the ED pathway on glucose consumption. To achieve this, we used a genetically modified *C. glutamicum* strain as a host strain that produces ethanol as the primary metabolite under conditions of oxygen deprivation. The reason we chose this strain was to avoid the involvement of NADPH in sugar metabolism, and to facilitate focusing on the influence of the “bypass effect” by the ED pathway on glycolysis. Furthermore, we investigated the influence of inactivation of the EMP pathway in an ED-introduced strain to verify whether the ED pathway alone is required for rapid glycolysis. Our findings suggested that coexistence of the ED and EMP pathways was needed to enhance the rate of glucose consumption; however, cofactors such as NADH and ATP were unlikely to be involved in enhanced glycolysis by the ED pathway.

## Results

### Pathway design

The ED pathway was introduced into the genome of the ethanol-producing *C. glutamicum* CRZ2e that was previously constructed by expressing the heterologous genes for *Z. mobilis* pyruvate decarboxylase and alcohol dehydrogenase from pCRA723 and disrupting the chromosomal genes for lactate dehydrogenase and phosphoenolpyruvate carboxylase to suppress the formation of lactate and succinate [21]. The initial reaction of the ED pathway is catalyzed by glucose-6-phosphate dehydrogenase (G6PDH) (Fig. 1). Although G6PDH from *C. glutamicum* is an NADP^+^-dependent enzyme, there is no enzyme that re-oxidizes NADPH in the ethanol production pathway of *C. glutamicum* CRZ2e, which results in an intracellular redox imbalance and slows down sugar metabolism. To circumvent the redox imbalance, we introduced the *zwf* gene from *Z. mobilis* encoding G6PDH because this enzyme accepts both NAD^+^ and NADP^+^ as cosubstrates. The *K*_*m*_ value of G6PDH from *Z. mobilis* is about 5 times higher for NADP^+^ than NAD^+^, but *V*_max_ is 1.7 times higher for NADP^+^ [22]. Enzymes 6-phosphogluconolactonase are also required for a functional ED pathway. For these enzymes to function, *pgl* (cgR_1628) from *C. glutamicum* encoding phosphogluconolactonase, and two *Z. mobilis* genes *edd* and *eda* encoding 6PGD and KDPGA, respectively, were simultaneously expressed in *C. glutamicum* CRZ2e. The resulting strain, designated as CRZ2e-ED, was expected to metabolize glucose through both the EMP and ED pathways. In addition, a strain with a disrupted *pfkA* gene was constructed using the CRZ2e-ED strain (designated as CRZ2e-EDΔ*pfkA*) that metabolizes glucose solely through the ED pathway.

### Functional expression of the ED pathway in *C. glutamicum*

Table 1 shows the activities of the selected enzymes in the recombinant strains. The activities of NAD^+^-dependent G6PDH, 6PGD, and KDPGA were detected in CRZ2e-ED and CRZ2e-EDΔ*pfkA*, but not in the parental CRZ2e strain. PFK activity was undetected in CRZ2e-EDΔ*pfkA* alone.

**Table 1:** Enzyme activities of recombinant *C. glutamicum* strains

Strain	Activity (U/mg protein)^a^			
	G6PDH^b^	6PGD	KDPGA	PFK
CRZ2e	ND^c^	ND^c^	ND^c^	0.17 ± 0.02
CRZ2e-ED	5.3 ± 0.1	0.83 ± 0.04	3.2 ± 0.1	0.09 ± 0.00
CRZ2e-EDΔ*pfkA*	6.3 ± 0.1	0.69 ± 0.07	3.4 ± 0.1	ND^c^

^a^Data show averages and standard deviations from three measurements

^b^NAD^+^ was used as a cofactor

^c^Activity less than 0.01 U/mg

**Fig. 2:**
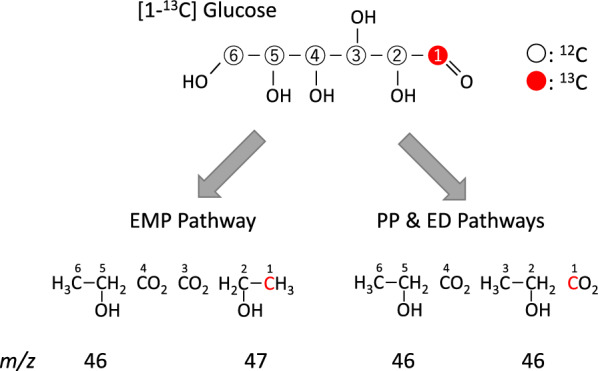
Mass isotopic distribution of ethanol from [1-^13^C] glucose metabolized through the EMP, ED, and PP pathways

To test whether the cells of the above recombinant strains had functional ED pathways, experiments on sugar metabolism were performed using ^13^C-labeled glucose. As shown in Fig. 2, [1-^13^C] glucose metabolism through the EMP pathway results in equal amounts of non-labeled and [2-^13^C] ethanol, while in the PP and ED pathways, the ^13^C label is removed as CO_2_ [23]. Mass isotopic distributions of ethanol produced from [1-^13^C] glucose by the recombinant strains were analyzed using GC–MS. Table 2 shows normalized intensities of mass fragments of *m/z* 46 and *m/z* 47 that are derived from the parent ions of non-labeled and [2-^13^C] ethanol, respectively. As expected, an obvious peak of *m/z* 47 was detected in the CRZ2e strain, but not in the ED-introduced strains, CRZ2e-ED and CRZ2e-EDΔ*pfkA*. A comparison between CRZ2e-ED and CRZ2e-EDΔ*pfkA* showed that the intensity of *m/z* 47 in CRZ2e-EDΔ*pfkA* was smaller than that in CRZ2e-ED (Tukey test, *p* < 0.05) and the intensity value in CRZ2e-EDΔ*pfkA* was as small as that observed for non-labeled ethanol. These findings suggest that glucose was at least partially metabolized via the ED pathway in the ED-introduced strains and the carbon flow to the EMP pathway was reduced by the introduction of the ED pathway and it was at negligible levels in the *pfkA* deletion strain.

**Table 2:** Mass isotopic distribution of ethanol produced from [1-^13^C] glucose by recombinant *C. glutamicum* strains

Strain	Normalized intensity of mass fragment^1^		Tukey test^2^
	*m/z* 46	*m/z* 47	
CRZ2e	41.6 ± 0.6	11.4 ± 0.6	a
CRZ2e-ED	27.4 ± 0.4	3.02 ± 3.02	b
CRZ2e-EDΔ*pfkA*	24.8 ± 0.2	0.85 ± 0.43	c
Non-labeled ethanol	23.8 ± 0.5	1.02 ± 0.02	c

Data show averages and standard errors from three measurements

^1^The intensity was normalized by the intensity of *m/z* 31

^2^The different letters (a–c) indicate significant differences in *m/z* 47 between the samples (one-way ANOVA followed by Tukey’s HSD test, *P* < 0.05)

### Comparison of glucose consumption and ethanol production under oxygen deprivation

Results of fermentation tests with the three recombinant strains are summarized in Table 3. The rates of sugar consumption and ethanol production of CRZ2e-ED were 1.5- and 2-fold higher than those of parental CRZ2e (*p* < 0.01), respectively. CRZ2e-EDΔ*pfkA* exhibited a rate of sugar consumption similar to CRZ2e; however, it showed a 1.6-fold higher ethanol production than CRZ2e, suggesting that the ethanol yield was improved in the CRZ2e-EDΔ*pfkA* strain. Along with the increase in the ethanol yield of CRZ2e-EDΔ*pfkA*, dihydroxyacetone and glycerol by-production by CRZ2e-EDΔ*pfkA* decreased less than one-twentieth of CRZ2e. The dihydroxyacetone and glycerol yields of CRZ2e-EDΔ*pfkA* were 6 and 2 mmol/mol of glucose consumed, respectively. These findings indicated that strains metabolizing glucose through a single glycolytic pathway (the EMP or the ED pathway) exhibited similar rate of glucose consumption and the CRZ2e-ED strain with two glycolytic pathways consumed glucose at a higher rate than the strains that metabolized glucose through a single pathway.

**Table 3:** Productivity and yield of ethanol of recombinant *C. glutamicum* strains in small-scale reaction

Strain	Rate (mmol/g-CDW/h)^1^		Ethanol yield (%)^1^
	Glucose consumption	Ethanol productivity	
CRZ2e	1.6 ± 0.1 a	2.1 ± 0.1 a	71 ± 2 a
CRZ2e-ED	2.4 ± 0.1 b	4.2 ± 0.2 b	91 ± 2 b
CRZ2e-EDΔ*pfkA*	1.8 ± 0.1 a	3.4 ± 0.1 c	100 ± 1 c

Data obtained from 2-h reactions show the average and the standard deviation from three independent experiments

^1^The percentage yield was calculated based on a theoretical maximum (0.51 g ethanol/g glucose consumed). The different letters (a–c) in the same column indicate significant differences between the samples (one-way ANOVA followed by Tukey’s HSD test, *P* < 0.05)

### The effects of ED pathway introduction on glucose consumption and cell growth under aerobic culture conditions

**Fig. 3:**
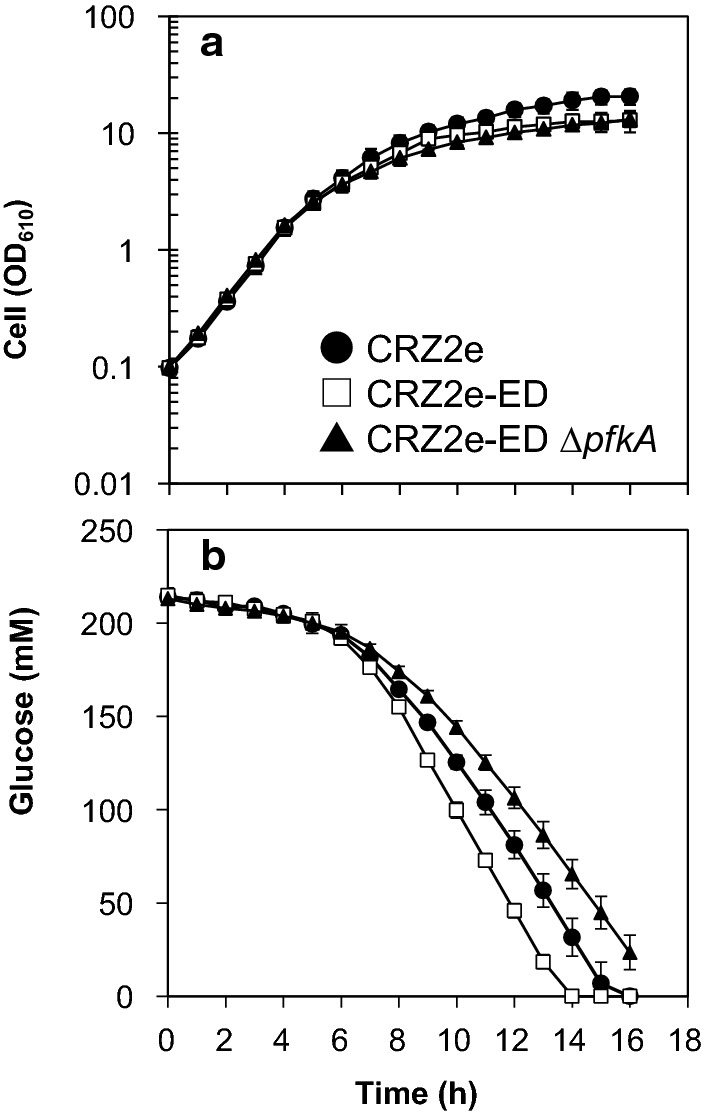
Cell growth (**a**) and glucose consumption profile (**b**) of CRZ2e, CRZ2e-ED, and CRZ2e-ED *∆pfkA* under aerobic conditions. Data represent averages and standard deviations from triplicate experiments

Figure 3 shows aerobic growth and glucose consumption of CRZ2e, CRZ2e-ED, and CRZ2e-EDΔ*pfkA* strains. Table 4 summarizes the specific rates of glucose consumption and cell growth calculated from Fig. 3. A significant difference in cell growth was not found between the three strains. On the other hand, the specific glucose consumption rate was the highest for the CRZ2e-ED strain and those of CRZ2e and CRZ2e-EDΔ*pfkA* were comparable. The cell yields were lower in the strains with the ED pathway than in CRZ2e. These results showed that strains coexisting with the ED and EMP pathways had the highest specific sugar consumption, similar to the results under oxygen deprivation.

**Table 4:** Glucose consumption and cell growth under aerobic culture conditions of recombinant *C. glutamicum* strains

Strain	μ (h^−1^)^1^	ν (g-glc/g-dry cell/h)^2^	Cell yield^3^
CRZ2e	0.73 ± 0.06 a	1.14 ± 0.11 a	0.20 ± 0.02 a
CRZ2e-ED	0.72 ± 0.06 a	1.78 ± 0.17 b	0.11 ± 0.01 b
CRZ2e-EDΔ*pfkA*	0.71 ± 0.05 a	1.10 ± 0.08 a	0.13 ± 0.02 b

The different letters (a, b) in the same column indicate significant differences between the samples (one-way ANOVA followed by Tukey’s HSD test, *P* < 0.05). The data are averages from three independent experiments

^1^Specific growth rate

^2^Specific glucose consumption rate

^3^Dry cell weight (g)/consumed glucose weight (g)

### Simultaneous overexpression of genes for the EMP and ED pathways

**Fig. 4:**
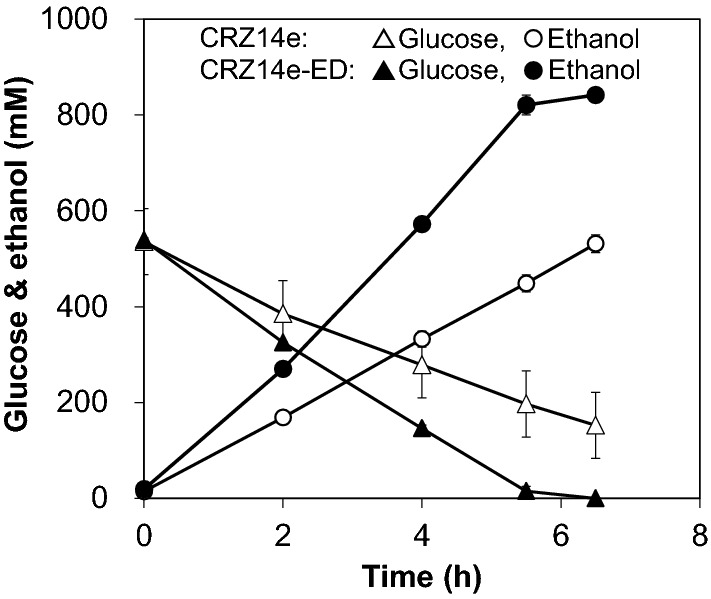
Ethanol production by *C. glutamicum* CRZ14e and CRZ14e-ED under conditions of oxygen deprivation. Data represent averages and standard deviations from triplicate experiments

To investigate whether the ED pathway further accelerates the sugar consumption rate also in the strain with enhanced expression of EMP pathway genes, the ED pathway was introduced to a previously constructed efficient ethanol producer, *C. glutamicum* CRZ14e, where five glycolytic genes (*pgi*, *pfkA*, *tpi*, *gapA*, and *pyk*) as well as *pdc* and *adhB* are simultaneously overexpressed [14]. Since a preliminary experiment revealed that the overexpression of *pgl* did not affect sugar consumption (data not shown), three *Z. mobilis* genes, *zwf*, *edd*, and *eda*, were integrated into the genome of CRZ14e to construct CRZ14e-ED. Figure 4 shows a comparison of ethanol production by CRZ14e and CRZ14e-ED. The rate of ethanol production and sugar consumption by CRZ14e-ED were 1.8- and 1.5-fold higher than those of the parental CRZ14e strain, respectively. The specific productivity and ethanol yield of CRZ14e-ED were 7.5 mmol/g-CDW/h and 92%, respectively. This productivity was also approximately 1.8-fold higher than that of CRZ2e-ED, where glycolytic genes are not overexpressed. These findings indicated that reinforcement of the ED and EMP pathways additively enhanced glucose consumption.

To gain insights into the mechanism of enhanced sugar consumption by ED pathway introduction, alterations of important coenzymes and intracellular metabolites induced by ED introduction were investigated. Intracellular metabolites of CRZ14e and CRZ14e-ED were determined after a 2-h reaction. Since CRZ14e-ED showed 1.8-fold faster productivity of ethanol than CRZ14e as described above, ethanol concentrations at the same reaction time must be largely different between CRZ14e-ED and CRZ14e, which probably affects the intracellular environment. To avoid this problem, half of the CRZ14e-ED cells were subjected to a reaction of ethanol production to analyze intracellular metabolites; almost equal amounts of ethanol are produced by CRZ14e and CRZ14e-ED (Additional file 1: Figure S1). Based on a comparison between the two strains, more than twofold differences in concentrations were found in NADPH and 6PG (Table 5). KDPG, the key metabolite in the ED pathway, was detected only in CRZ14e-ED, which indicated the presence of a functional ED pathway and parts of G6P was directed to the ED pathway in CRZ14e-ED. Intracellular NADH and ATP are important for the control of sugar metabolism. While ATP concentrations showed small differences between these strains, almost no change was observed in the NADH/NAD^+^ ratio.

**Table 5:** A comparison of intracellular metabolites in CRZ14e and CRZ14e-ED

Metabolite	Intracellular metabolite (mM)		Ratio	*P* value^a^
	CRZ14e	CRZ14e-ED		
G6P	0.22 ± 0.01	0.25 ± 0.02	1.1	0.04
F6P	0.03 ± 0.00	0.03 ± 0.01	1.0	0.27
FBP	34 ± 3	38 ± 2	1.1	0.12
DHAP	3.4 ± 0.1	3.7 ± 0.2	1.1	0.03
GAP	0.16 ± 0.01	0.23 ± 0.03	1.4	0.02
BPG	0.04 ± 0.02	0.08 ± 0.01	2.0	0.02
3PG	0.72 ± 0.08	1.15 ± 0.06	1.6	0.00
PEP	0.12 ± 0.00	0.17 ± 0.02	1.4	0.02
PYR	2.18 ± 0.41	2.29 ± 0.13	1.1	0.68
Ru5P	0.19 ± 0.01	0.12 ± 0.01	0.6	0.00
R5P	0.09 ± 0.01	0.07 ± 0.00	0.8	0.02
6PG	0.03 ± 0.00	0.09 ± 0.01	3.0	0.00
ADP	0.37 ± 0.02	0.32 ± 0.03	0.9	0.07
ATP	1.70 ± 0.05	1.92 ± 0.10	1.1	0.02
NAD	0.28 ± 0.03	0.37 ± 0.07	1.3	0.14
NADH	0.07 ± 0.00	0.07 ± 0.02	1.0	0.97
NADP	0.17 ± 0.02	0.11 ± 0.01	0.6	0.01
NADPH	0.02 ± 0.01	0.10 ± 0.01	5.0	0.00
KDPG	0.00 ± 0.00	0.09 ± 0.01	NA	0.00
ATP/ADP	4.64 ± 0.29	5.98 ± 0.49	1.3	0.02
NADH/NAD	0.24 ± 0.02	0.19 ± 0.09	0.8	0.45

Data obtained from 2-h reactions show the average and the standard deviation from three measurements

^a^Student’s *t* test was used for statistical analyses

## Discussion

In the present study, we revealed that the introduction of the ED pathway to *C. glutamicum* enhanced glucose consumption under conditions of oxygen deprivation and, specifically, we showed that the coexistence of the ED and EMP pathways is crucial to improve glucose consumption, which indicates the importance of the bypass effect of the ED pathway. In addition, we showed that inducing the function of the ED and EMP pathways is a good strategy for improving biocatalyst productivity even when NADPH supply is not a limiting factor for fermentation.

Findings from experiments conducted using ^13^C-labeled glucose suggested that glucose is partially metabolized through the ED and/or PP pathway in the ED-introduced strains. The question then arises whether the ED or the PP pathway dominates sugar metabolism in those strains. The value of NADPH/NADP^+^ in CRZ14e-ED was 6.3-fold higher than that of the parental strain CRZ14e. Since G6PDH from *Z. mobilis* introduced in CRZ14e-ED accepts both NADP^+^ and NAD^+^ as cosubstrates [24], we speculated that both NADPH and NADH must be produced through glucose metabolism. However, only NADH is re-oxidized by reducing acetaldehyde to ethanol in the *C. glutamicum* strains, where the reaction is catalyzed by NADH-dependent alcohol dehydrogenase, resulting in high NADPH/NADP^+^. 6PGDH in the PP pathway of *C. glutamicum* is inhibited by NADPH [25]; therefore, the carbon flow to the PP pathway would be strongly suppressed in the CRZ14e-ED strain because of a high NADPH/NADP^+^ ratio (Fig. 1). Therefore, we speculated that carbon flow to the PP pathway is limited immediately after the reaction starts and subsequently glucose is metabolized through the ED and EMP pathways in the ED-introduced strains of *C. glutamicum*.

*Z. mobilis* exhibits high rates of glucose consumption and ethanol production [18]. The ATP yield from glycolysis in the ED pathway is half that of the EMP pathway; therefore, *Z. mobilis* is believed to consume more glucose to compensate for the lower ATP yield [26]. The CRZ2e-EDΔ*pfkA* strain relies solely on the ED pathway for glycolysis; however, it showed a lower rate of sugar consumption than the CRZ2e-ED strain with functional ED and EMP pathways (Table 3) and the ATP/ADP ratio of the CRZ14e-ED strain was slightly higher than that of the CRZ14e strain (Table 5). Furthermore, if ATP controls the sugar consumption of *C. glutamicum* under conditions of oxygen deprivation, the sugar consumption rate of CRZ2e-EDΔ*pfkA*, which metabolizes sugar only through the ED pathway, should be faster than that of the CRZ2e and CRZ2e-ED strains, considering the fact that the ATP yield from the ED pathway is half that of the EMP pathway. However, CRZ2e-EDΔ*pfkA* showed a similar or slower rate of sugar consumption compared to the other strains, which indicated that ATP is not involved in controlling the glycolytic flux of *C. glutamicum* under conditions of oxygen deprivation. These findings are not consistent with those of *E. coli*, where overexpression of the ATP-consuming enzyme (H^+^-ATP synthase) increases the rate of sugar consumption [27]. It is noteworthy that the ATP/ADP ratio of *E. coli* reported by Koebmann et al. was > 10, which is much higher than 4.64 and 5.98 for the CRZ14e and CRZ14e-ED strains, respectively (Table 5). The ATP/ADP ratio observed in the present study is possibly below the threshold affecting sugar consumption, and therefore, its influence on sugar consumption might be small in the present study.

GAPDH is one of the main enzymes controlling glycolytic flux in *C. glutamicum* under conditions of oxygen deprivation [8, 10]. Half of the carbons derived from glucose bypass the reaction catalyzed by GAPDH in the ED pathway, which could explain the increased sugar consumption observed in this study. Very recently, Liang et al. reported that the ED pathway from *Z. mobilis* artificially constructed in a butanol-producing *E. coli* resulted in the improvement of butanol productivity and glucose consumption [17], even though *E. coli* has an endogenous ED pathway. Findings from our study and Liang’s study showed that the parallel functioning of the EMP and ED pathways in a single cell enhances glucose consumption. These observations gave rise to a simple question: does the introduction of the EMP pathway in *Z. mobilis* improve glucose consumption and ethanol productivity? Chen et al. investigated the overexpression of PFK, the missing enzyme for the EMP pathway in *Z. mobilis*, to study the sugar metabolism in *Z. mobilis*; however, they failed to redirect the carbon flux from the ED to the EMP pathway in *Z. mobilis*, possibly owing to a rigid regulation mechanism of the ED pathway in this microbe [28]. Therefore, studies that focus on developing a genetic engineering strategy for the coexistence of sugar metabolic pathways in a single cell are required.

## Conclusions

In the present study, we revealed that coexistence of the ED and EMP pathways was required to enhance the rate of glucose consumption; however, cofactors such as NADH and ATP were unlikely to be involved in enhanced glycolysis. Thus, coexistence of the ED and EMP pathways is a good strategy for improving biocatalyst productivity even when NADPH supply is not a limiting factor for fermentation. Based on these findings, we constructed the recombinant strain CRZ14e-ED with simultaneous overexpression of the genes for the EMP and ED pathways. CRZ14e-ED exhibited the highest ethanol productivity among all *C. glutamicum* strains ever constructed.

## Materials and methods

### Microbial strains and growth media

All strains listed in Table 6 were derived from the *C. glutamicum* strain R (JCM 18229) [29] and they were grown in nutrient-rich A-medium containing 40 g/L glucose [6]. Where appropriate, the medium was supplemented with chloramphenicol (5 mg/L).

**Table 6:** Strains and plasmids used in this study

Strain and plasmid	Genotype or description	Reference
*C. glutamicum*		
R	Wild-type (JCM 18229)	[29]
CRZ2	R with deletion in *ldhA* and *ppc*	[21]
Gly3	CRZ2 with insertion of P*tac*-*pgi*, P*tac*-*pfkA*, P*tac*-*gapA* and P*tac*-*pyk*	[12]
CRZ2e	CRZ2 harboring pCRA723	[21]
CRZ14	Gly3 with insertion of P*tac*-*tpi*	[14]
CRZ14e	CRZ14 with overexpression of *pdc* and *adhB* from pCRA723	[14]
CRZ2e-ED	CRZ2e with insertion of P*tac*-*zwf*, P*tac*-*pgl*, P*tac*-*edd*-*eda*	This study
CRZ2e-EDΔ*pfkA*	CRZ2e-ED with deletion in *pfkA*	This study
CRZ14e-ED	CRZ14e with insertion of P*tac*-*zwf*, P*tac*-*pgl*, P*tac*-*edd-eda*	This study
Plasmid		
pCRA723	Cm^r^; *E. coli*–*Corynebacterium* sp. shuttle vector derived from pBL1 with *Z. mobilis pdc* and *adhB*	[21]

### Genetic engineering

Chromosomal DNA was isolated from *Z. mobilis* ZM4 and used as PCR template to amplify *eda*, *zwf*, and *edd*. Primers used in the present study are listed in Additional file 2: Table S1. PCR was performed using a GeneAmp PCR 9700 system (Applied Biosystems, USA) and PrimeStar HS DNA polymerase (Takara, Japan). The *tac* promoter was fused to the 5′ end of each gene and the resulting DNA fragments were integrated into *C. glutamicum* R strain-specific islands in the chromosome, as described previously [30]. Markerless *pfkA* disruption was performed according to a method described previously, which is based on homologous recombination, followed by *sacB* selection [21]. Gene disruption was confirmed by PCR. Transformation of *C. glutamicum* was performed by electroporation by delivering an electrical pulse of 2.5 kV, 200 Ω resistance, and 25 µF capacitance in a 0.1-cm cuvette using a Gene Pulser apparatus (Bio-Rad, USA).

### Enzyme assay

Preparation of crude extracts from cells was conducted according to the previous report [6]. Protein concentrations were measured using a protein assay kit (Bio-Rad, USA). G6PDH activity was measured by the method described previously [22]. An assay mixture contained 1 mM NAD^+^, 1 mM glucose-6-phosphate, 30 mM KCl and 2 mM MgCl_2_ in 50 mM Tris–HCl buffer, pH 8. The reaction was started by the addition of appropriate amount of the crude extracts to the assay mixture. 6PGD and KDPGA activities were determined by the method described previously [31]. An assay mixture for 6PGD contained cell extract, 8 mM 6-phosphogluconate, 10 mM MgCl_2_ in 50 mM Tris–HCl buffer, pH 7.6. After 5 min at 30 °C, the reaction mixture was diluted by the same buffer to 2 mL and heated for 2 min at 100 °C. After centrifugation, the supernatant was measured for pyruvate with 2 U lactate dehydrogenase and 0.4 mM NADH. For measurement of KDPGA activity, a reaction mixture contained cell extract, 0.1 mM KDPG, 10 mM MgCl_2_ in 50 mM Tris–HCl buffer, pH 7.6. After 5 min at 30 °C, pyruvate was measured by the same method as 6PGD. For measurement of PFK activity, an assay mixture contained 5 mM fructose-6-phosphate, 4 mM MgCl_2_, 5 mM dithiothreitol, 1 U aldolase, 1 U triosephosphate isomerase, 1 U GAPDH, 1 mM ATP, and 0.2 mM NADH in 50 mM Tris–HCl buffer, pH 8.0.

### Conversion reaction under oxygen deprivation

*C. glutamicum* strains were aerobically cultivated at 33 °C for 16–20 h in a 2-L flask containing 500 mL of A-medium, supplemented with 40 g/L glucose. Cells were harvested by centrifugation (5000 × *g*, 4 °C, 10 min), washed once, and resuspended in minimal salts medium [6]. The reaction temperature was maintained at 33 °C. Oxygen deprivation conditions (dissolved oxygen concentration < 0.01 parts per million) were achieved with high cell density, no aeration, and gentle agitation. The pH was monitored using a pH controller (DT-10023, Biott Co., Japan) and maintained at pH 6.5 by supplementation with 2.5 M ammonium hydroxide. The percentage yield of ethanol from glucose was calculated based on a theoretical maximum (0.51 g ethanol/g sugar consumed). For a small-scale reaction, the washed cells resuspended in the minimal salts medium were mixed with an equal volume of 200 mM MES buffer (pH 6.5) containing 100 mM glucose to start a reaction in a total reaction volume of 10 mL. The pH of the reaction mixture was maintained in the range of pH 6.4–6.5 during the 2-h reaction at 33 °C.

### Determination of mass isotopic distribution

In experiments using ^13^C-labeled glucose, the washed cells were resuspended in 0.5 mL of the minimal salts medium, and an equal amount of 200 mM MES buffer (pH 6.5) containing 10 mM [1-^13^C] glucose (Cambridge Isotope Laboratories, Inc., USA) was added to the cell suspension to start a reaction. After 2 h, the supernatant was recovered for GC–MS analysis.

### Analytical techniques

The ethanol concentration was determined using a gas chromatogram (GC2014, Shimadzu, Japan) equipped with a Thermon-1000 Sunpak-A 50/80 (Shinwa Chemical Industries, Japan). Glucose concentration was determined using HPLC (model 8020, Tosoh, Japan) as described previously [14]. In experiments using ^13^C-labeled glucose, ethanol was analyzed using GC–MS (QP2010 system, Shimadzu, Japan) equipped with a capillary column of DB-5MS (30 m × 0.25 μm id; Agilent Technologies, Santa Clara, CA, USA). The injector temperature was set at 230 °C and 1 μl of the supernatant was injected in split mode (1:30). The GC was operated at a constant flow of 1 mL min^−1^ helium. The temperature was held constant at 40 °C for 7 min.

### Metabolome analysis

Intracellular metabolites were extracted from *C. glutamicum* cells as follows. Reaction mixture containing the cells (25 μl) was taken 2 h after the conversion reaction started and immediately quenched by mixing with 1.0 ml cold methanol (− 80 °C). The resultant cell suspension (0.5 ml) was mixed vigorously with 0.5 ml chloroform and 0.5 ml H_2_O (− 20 °C), and after being incubated for 60 min at − 20 °C, the sample solution was centrifuged (20,000 × *g*, 4 °C; 5 min) and the upper layer (50 μl) was mixed with 50 μl H_2_O or authentic standard mixture solution (5.0 μM each). The resultant supernatant was analyzed using HPLC (Prominence 20A; Shimadzu) coupled with a linear ion trap mass spectrometer (4000 Q TRAP; Applied Biosystems/MDS Sciex, USA). Intracellular metabolites were analyzed by ion-pairing reversed-phase liquid chromatography with 5 mM dibutylammonium acetate (Tokyo Chemical Industry, Japan) as described previously [32]. Statistical analysis of the data was performed using one-way ANOVA with Tukey’s test and Student’s *t* test.

## Supplementary Information

**Additional file 1: Figure S1.** Ethanol production and glucose consumption by CRZ14e and CRZ14e-ED for the metabolome analysis of Table 5. Data represent averages and standard deviations from triplicate experiments.

**Additional file 2: Table S1.** Primers used in this study.

## Data Availability

Data will be made available from the corresponding author on reasonable request.

## Abbreviations

3PG: 3-Phosphoglycerate
6PG: 6-Phosphogluconate
6PGD: 6-Phosphogluconate dehydratase
6PGDH: 6-Phosphogluconate dehydrogenase
BPG: 1,3-Bisphosphoglycerate
DHAP: Dihydroxyacetone phosphate
E4P: Erythrose 4-phosphate
ED pathway: Entner–Doudoroff pathway
EMP pathway: Embden–Meyerhof–Parnas pathway
F6P: Fructose 6-phosphate
FBA: Fructose-bisphosphate aldolase
FBP: Fructose 1,6-bisphosphate
G6P: Glucose-6-phosphate
GAP: Glyceraldehyde-3-phosphate
G6PDH: Glucose-6-phosphate dehydrogenase
GAPDH: Glyceraldehyde-3-phosphate dehydrogenase
KDPG: 2-Keto-3-deoxy-6-phosphogluconate
KDPGA: 2-Keto-3-deoxy-6-phosphogluconate aldolase
PEP: Phosphoenolpyruvate
PFK: 6-Phosphofructokinase
PP pathway: Pentose phosphate pathway
PYK: Pyruvate kinase
PYR: Pyruvate
R5P: Ribose 5-phosphate
Ru5P: Ribulose 5-phosphate

## References

[1] Davy AM, Kildegaard HF, Andersen MR (2017). Cell factory engineering. Cell Syst.

[2] Jojima T, Inui M (2015). Engineering the glycolytic pathway: a potential approach for improvement of biocatalyst performance. Bioengineered.

[3] Hauf J, Zimmermann FK, Müller S (2000). Simultaneous genomic overexpression of seven glycolytic enzymes in the yeast *Saccharomyces cerevisiae*. Enzyme Microb Technol.

[4] Schaaff I, Heinisch J, Zimmermann FK (1989). Overproduction of glycolytic enzymes in yeast. Yeast.

[5] Hasegawa S, Tanaka Y, Suda M, Jojima T, Inui M (2017). Enhanced glucose consumption and organic acid production by Engineered *Corynebacterium glutamicum* based on analysis of a *pfkB1* deletion mutant. Appl Environ Microbiol.

[6] Jojima T, Fujii M, Mori E, Inui M, Yukawa H (2010). Engineering of sugar metabolism of *Corynebacterium glutamicum* for production of amino acid L-alanine under oxygen deprivation. Appl Microbiol Biotechnol.

[7] Ikeda M, Takeno S, Yukawa H, Inui M (2013). Amino acid production by *Corynebacterium glutamicum*. Corynebacterium glutamicum.

[8] Inui M, Murakami S, Okino S, Kawaguchi H, Vertès AA, Yukawa H (2004). Metabolic analysis of *Corynebacterium glutamicum* during lactate and succinate productions under oxygen deprivation conditions. J Mol Microbiol Biotechnol.

[9] Jojima T, Inui M, Yukawa H, Yukawa HIM (2013). Biorefinery applications of *Corynebacterium glutamicum*. Corynebacterium glutamicum.

[10] Tsuge Y, Uematsu K, Yamamoto S, Suda M, Yukawa H, Inui M (2015). Glucose consumption rate critically depends on redox state in *Corynebacterium glutamicum* under oxygen deprivation. Appl Microbiol Biotechnol.

[11] Hasegawa S, Suda M, Uematsu K, Natsuma Y, Hiraga K, Jojima T (2013). Engineering of *Corynebacterium glutamicum* for high-yield L-valine production under oxygen deprivation conditions. Appl Env Microbiol.

[12] Yamamoto S, Gunji W, Suzuki H, Toda H, Suda M, Jojima T (2012). Overexpression of genes encoding glycolytic enzymes in *Corynebacterium glutamicum* enhances glucose metabolism and alanine production under oxygen deprivation conditions. Appl Env Microbiol.

[13] Tsuge Y, Yamamoto S, Kato N, Suda M, Vertès AA, Yukawa H (2015). Overexpression of the phosphofructokinase encoding gene is crucial for achieving high production of D-lactate in *Corynebacterium glutamicum* under oxygen deprivation. Appl Microbiol Biotechnol.

[14] Jojima T, Noburyu R, Sasaki M, Tajima T, Suda M, Yukawa H (2015). Metabolic engineering for improved production of ethanol by *Corynebacterium glutamicum*. Appl Microbiol Biotechnol.

[15] Okahashi N, Matsuda F, Yoshikawa K, Shirai T, Matsumoto Y, Wada M (2017). Metabolic engineering of isopropyl alcohol-producing *Escherichia coli* strains with ^13^C-metabolic flux analysis. Biotechnol Bioeng.

[16] Nagai H, Masuda A, Toya Y, Matsuda F, Shimizu H (2018). Metabolic engineering of mevalonate-producing *Escherichia coli* strains based on thermodynamic analysis. Metab Eng Academic Press.

[17] Liang S, Chen H, Liu J, Wen J (2018). Rational design of a synthetic Entner-Doudoroff pathway for enhancing glucose transformation to isobutanol in *Escherichia coli*. J Ind Microbiol Biotechnol.

[18] Rogers PL, Lee KJ, Skotnicki ML, Tribe DE (1982). Ethanol production by *Zymomonas mobilis*.

[19] Okino S, Inui M, Yukawa H (2005). Production of organic acids by *Corynebacterium glutamicum* under oxygen deprivation. Appl Microbiol Biotechnol.

[20] Radoš D, Turner DL, Fonseca LL, Carvalho AL, Blombach B, Eikmanns BJ (2014). Carbon flux analysis by ^13^C nuclear magnetic resonance to determine the effect of CO_2_ on anaerobic succinate production by *Corynebacterium glutamicum*. Appl Environ Microbiol.

[21] Inui M, Kawaguchi H, Murakami S, Vertès AA, Yukawa H (2004). Metabolic engineering of *Corynebacterium glutamicum* for fuel ethanol production under oxygen-deprivation conditions. J Mol Microbiol Biotechnol.

[22] Scopes RK, Testolin V, Stoter A, Griffiths-Smith K, Algar EM (1985). Simultaneous purification and characterization of glucokinase, fructokinase and glucose-6-phosphate dehydrogenase from *Zymomonas mobilis*. Biochem J Biochem J.

[23] Bayle K, Akoka S, Remaud GS, Robins RJ (2015). Nonstatistical ^13^C distribution during carbon transfer from glucose to ethanol during fermentation is determined by the catabolic pathway exploited. J Biol Chem.

[24] Scopes RK, Testolin V, Stoter A, Griffiths-Smith K, Algar EM (1985). Simultaneous purification and characterization of glucokinase, fructokinase and glucose-6-phosphate dehydrogenase from *Zymomonas mobilis*. Biochem J.

[25] Moritz B, Striegel K, de Graaf AA, Sahm H (2000). Kinetic properties of the glucose-6-phosphate and 6 phosphogluconate dehydrogenases from *Corynebacterium glutamicum* and their application for predicting pentose phosphate pathway flux *in vivo*. Eur J Biochem.

[26] Conway T (1992). The Entner-Doudoroff pathway: history, physiology and molecular biology. FEMS Microbiol Lett.

[27] Koebmann BJ, Westerhoff HV, Snoep JL, Nilsson D, Jensen PR (2002). The glycolytic flux in *Escherichia coli* is controlled by the demand for ATP. J Bacteriol.

[28] Chen RR, Agrawal M, Mao Z (2013). Impact of expression of EMP enzymes on glucose metabolism in *Zymomonas mobilis*. Appl Biochem Biotechnol.

[29] Yukawa H, Omumasaba CA, Nonaka H, Kos P, Okai N, Suzuki N (2007). Comparative analysis of the *Corynebacterium glutamicum* group and complete genome sequence of strain R. Microbiology.

[30] Sasaki M, Jojima T, Inui M, Yukawa H (2008). Simultaneous utilization of d-cellobiose, d-glucose, and d-xylose by recombinant *Corynebacterium glutamicum* under oxygen-deprived conditions. Appl Microbiol Biotechnol.

[31] Fraenkel DG, Horecker BL (1964). Pathways of d-glucose metabolism in *Salmonella typhimurium*: a study of a mutant lacking phosphoglucose isomerase. J Biol Chem.

[32] Ehira S, Shirai T, Teramoto H, Inui M, Yukawa H (2008). Group 2 sigma factor SigB of *Corynebacterium glutamicum* positively regulates glucose metabolism under conditions of oxygen deprivation. Appl Environ Microbiol.

