# Supplementary material for: Coexistence of the Entner–Doudoroff and Embden–Meyerhof–Parnas pathways enhances glucose consumption of ethanol-producing Corynebacterium glutamicum
Source: Biotechnol Biofuels. 2021 Feb 16;14:45. doi: 10.1186/s13068-021-01876-3 (PMC7888142; doi:10.1186/s13068-021-01876-3)
Supplement: Supplementary file 1 — Additional file 1: Figure S1. Ethanol production and glucose consumption by CRZ14e and CRZ14e-ED for the metabolome analysis of Table 5. Data represent averages and standard deviations from triplicate experiments. [file 13068_2021_1876_MOESM1_ESM.pptx]

## Slide 1
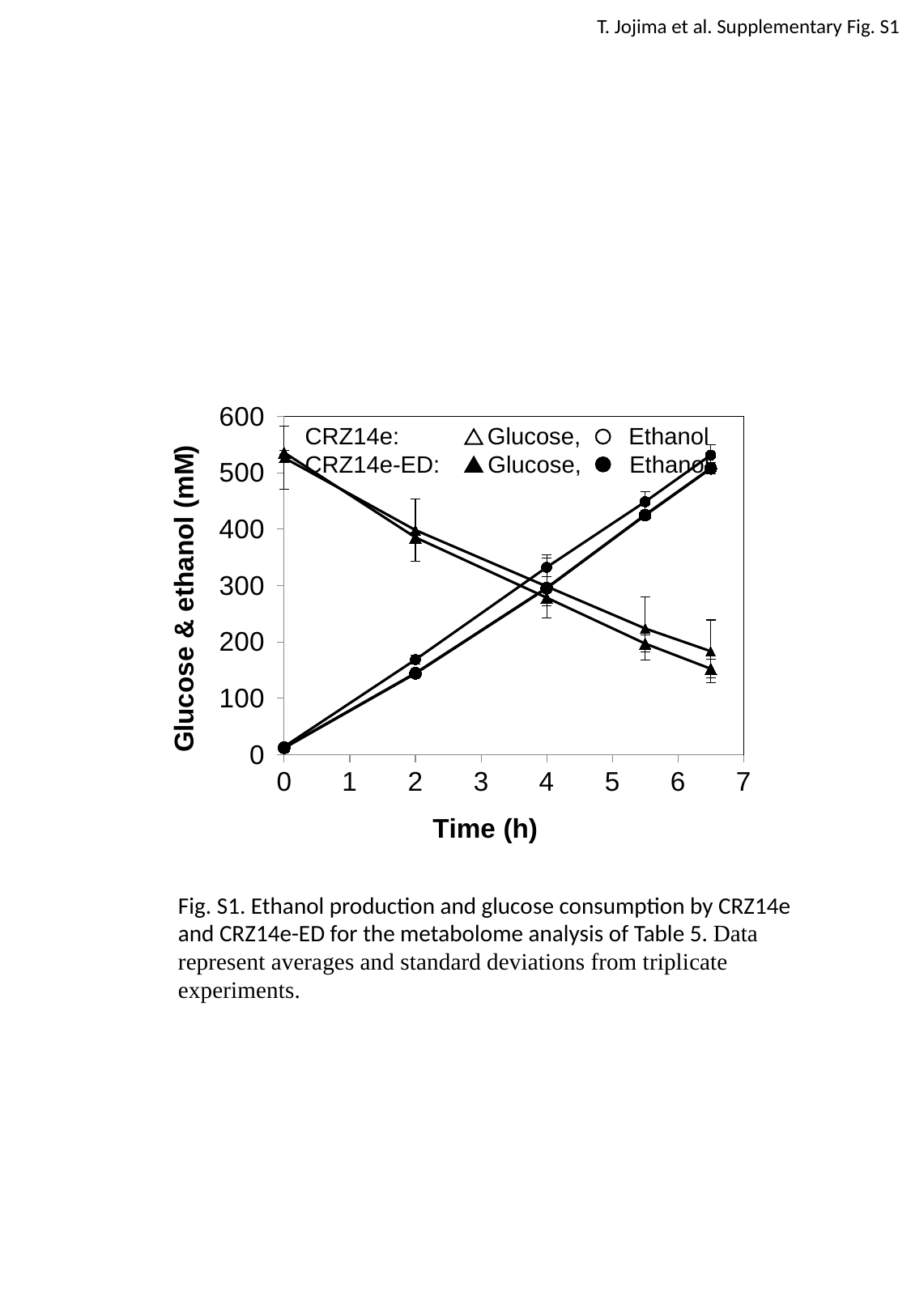

T. Jojima et al. Supplementary Fig. S1
### Chart
| Category | LPglc7 10% | LPglc186 5% | LPglc7 10% | LPglc186 5% |
|---|---|---|---|---|CRZ14e: 　 Glucose, 　 Ethanol
CRZ14e-ED: 　 Glucose, 　 Ethanol
Fig. S1. Ethanol production and glucose consumption by CRZ14e and CRZ14e-ED for the metabolome analysis of Table 5. Data represent averages and standard deviations from triplicate experiments.
