# Supplementary material for: Coexistence of the Entner–Doudoroff and Embden–Meyerhof–Parnas pathways enhances glucose consumption of ethanol-producing Corynebacterium glutamicum
Source: Biotechnol Biofuels. 2021 Feb 16;14:45. doi: 10.1186/s13068-021-01876-3 (PMC7888142; doi:10.1186/s13068-021-01876-3)
Supplement: Supplementary file 2 — Additional file 2: Table S1. Primers used in this study. [file 13068_2021_1876_MOESM2_ESM.docx]

Table S1. Primers used in this study

| Primer NO. | Sequence (5' to 3') | Target |
| --- | --- | --- |
| 1 | CTCTCATATGCGTGATATCGATTCCGTAATG | *eda* and SSI8 amplification |
| 2 | CTCTCATATGTTAGGCAACAGCAGCGC |  |
| 3 | CTCTCCTGCAGGCACGAACCTCAATTAGCCTG |  |
| 4 | CTCTCCTGCAGGGATGACTTGATGCAGGTGTG |  |
| 5 | CTCTAGTACTATGACAAATACCGTTTCGACGATG | *zwf-edd* and SSI3 amplification |
| 6 | CTCTAGTACTTTAGATACCGGCACCTGCAT |  |
| 7 | CTCTTCTAGAGCTTTGTTAGGTGTCTCTGG |  |
| 8 | CTCTTCTAGATCACCACCATGAAGAAGTCC |  |
| 9 | CTCTCTCGAGGCAACAGTGCTTCATACTGC |  |
| 10 | CTCTCTCGAGACGGATTGAACCCAAGACG |  |
| 11 | CTCTCCATGGTTGATGTAGTACGCGC | *pgl* and SSI6 amplification |
| 12 | CTCTCCATGGTTAGAGATTTCCTGCAGCATCATC |  |
| 13 | CTCTTCTAGAAGACATCGGAGCAATCGGCT |  |
| 14 | CTCTTCTAGAGTCCGCAGAGGAACCATTCA |  |
| 15 | CTCTGCATGCAGAAGAACTCGTGCTCAGC | *pfkA* deletion |
| 16 | GTAGCAATTCGCATGTCTTCC |  |
| 17 | GTTTAATATGGAAGACATGCGAATTGCTACGAAGGAAGTTCCATTCGAGC |  |
| 18 | CTCTGTCGACAATAGACAGATGCTCCGACG |  |

*SSI, strain specific island
